# Prevalence of Overweight, Obesity, and Abdominal Obesity in a Representative Sample of Portuguese Adults

**DOI:** 10.1371/journal.pone.0047883

**Published:** 2012-10-31

**Authors:** Luís B. Sardinha, Diana A. Santos, Analiza M. Silva, Manuel J. Coelho-e-Silva, Armando M. Raimundo, Helena Moreira, Rute Santos, Susana Vale, Fátima Baptista, Jorge Mota

**Affiliations:** 1 Exercise and Health Laboratory, Interdisciplinary Center for the Study of Human Performance, Faculty of Human Kinetics, Technical University of Lisbon, Cruz-Quebrada, Portugal; 2 Faculty of Sport Sciences and Physical Education, University of Coimbra, Coimbra, Portugal; 3 Research Center in Sports, Health Sciences and Human Development, Department of Sport and Health, University of Évora, Évora, Portugal; 4 Department of Sport Sciences, Exercise and Health, University of Trás-os-Montes and Alto Douro, Vila Real, Portugal; 5 Research Centre in Physical Activity, Health and Leisure, Faculty of Sports, University of Porto, Porto, Portugal; 6 Maia Institute of Higher Education, Maia, Portugal; University of Granada, Spain

## Abstract

This study determined the prevalence of overweight, obesity, and abdominal obesity in the Portuguese adults and examined the relationship between above mentioned prevalences and educational level. Body mass, stature, and waist circumference were measured in a representative sample of the Portuguese population aged 18–103 years (n = 9,447; 18–64 years: n = 6,908; ≥65 years: n = 2,539). Overweight and obesity corresponded to a body mass index ranging between 25–29.9 kg/m^2^ and ≥30 kg/m^2^, respectively. Abdominal obesity was assessed as >102 cm for males and >88 cm for females. After adjusting for educational level, the combined prevalences of overweight and obesity were 66.6% in males and 57.9% in females (18–64 years). Respective values in older adults (≥65 years) were 70.4% for males and 74.7% for females. About 19.3% of adult males and 37.9% of adult females presented abdominal obesity. Correspondent values in older adults were 32.1%, for males, and 69.7%, for females. In adults, low educational level was related to an increased risk for overweight (OR = 2.54; 95% CI: 2.08–3.09), obesity (OR = 2.76; 95% CI: 2.20–3.45), and abdominal obesity (OR = 5.48; 95% CI: 4.60–6.52). This reinforces the importance of adjusting public health strategies for educational level.

## Introduction

Total body and abdominal obesity (AO) have well-known associations with all-cause mortality [1], [2], morbidity [3] and disability, resulting in unhealthy life-years with poor quality of life [4], [5] and increased health care costs [6], [7]. To tackle this disease, nations have adopted preventive strategies and national campaigns. In order to better understand global, regional and national relevance of this disease over time, trends in prevalence data is critical to assess the effectiveness of these strategies and campaigns. Although a possible leveling off in the obesity prevalence has recently been discussed, in the European adult population, ambiguous data has been observed and, in some countries, increases are still reported in the adult population [8].

Prior studies have reported the prevalence of overweight and obesity in the Portuguese population [9], [10], [11] with both self-reported [11] and objectively measured weight and height [9]. The use of self-reported measures can lead to underestimation of overweight and obesity, as subjects tend to underestimate their weight and overestimate their height [12], [13]. However, even when using objectively measures of weight and height, no studies have adjusted for educational-related inequalities in overweight and obesity, in this population [9].

There is evidence that less educated adults are more likely to be obese than college graduates, the differentials are larger for women than men, and are more evident among younger adults [14], [15]. Similar observations have been reported for the Portuguese population with self-reported weight and height [11]. Understanding educational differences in weight status is important because it is not only about the income available for purchasing health-related goods and services, but are also about to cognitive skills and access to information that may affect health behavior [14]. Therefore, in order to present more accurate data, studies regarding the prevalence of obesity in population should consider differences related to educational inequalities.

To our knowledge no study has been conducted in Portugal, regarding the prevalence of overweight, obesity and AO, in adults aged 18 and older, using objective measures of weight, height and WC and considering the potential role of educational inequalities. Therefore, the aims of this study were (i) to report the prevalence of overweight, obesity and AO of the Portuguese adult population, using a representative sample; (ii) and to analyze how the educational level influences current overweight, obesity and AO in adult men and women.

## Materials and Methods

### Ethics Statement

All participants were informed about the objectives of the study and gave their written consent. The study was approved by the Ethics Committee attached to the scientific board of the Faculty of Human Kinetics at the Technical University of Lisbon and was conducted in accordance with the declaration of Helsinki for human studies of the World Medical Association [16].

### Participants

Data for the present study were derived from a cross sectional representative sample and a sampling frame with the Portuguese noninstitutionalized population aged 18 or more years in the five administrative and geographical regions of continental Portugal (Alentejo, Algarve, Centro, Lisboa, and Norte). Selection from sampling frame and data collection followed a multi-stage proportionate stratified cluster sampling procedure, considering population gender, age and geographical distribution. First, the sample was stratified by geographical region and size of municipality. Second, persons in households, work sites, and community settings within each municipality were randomly interviewed. Controlled quota sampling was calculated taking into account the demographic data reported by National Census. The survey had an overall response rate of about 80%. A total of 9,447 participants were evaluated in 2008 and 2009. The sample was divided into adults, aged 18–64 years old (n = 6,908), and old adults, aged 65 and older (n = 2,539). All the participants had independent physical function.

### Anthropometric Measurements

Participants were weighed to the nearest 0.1 kg wearing minimal clothes and without shoes and height was measured to the nearest 0.1 cm, according to the standardized procedures, as described elsewhere [17] with a scale coupled with a stadiometer (Seca, Hamburg, Germany).

Body mass index was calculated as weight (kg) divided by the square of the height (m) and classified into normal (<25 kg/m^2^), overweight (25–29.9 kg/m^2^) or obesity (≥30 kg/m^2^) [18].

Waist circumference was measured with a tape (Seca, Hamburg, Germany) at minimal respiration at the end of a normal expiration and reported to the nearest 0.1 cm by positioning a flexible anthropometric tape parallel to the floor and immediately above the iliac crest, according to NIH procedures used in the third U.S. National Health and Nutrition Examination Survey (NHANES III 1988–1994) protocol [19]. Waist circumference was then dichotomized into normal or increased risk (males>102 cm; females: >88 cm) [18].

### Educational Level

Participants were asked to report their educational level, according to the Portuguese educational system: Under 4 years, 5–9 years, 10–12 years, and higher education level. For data adjustment, a weight factor was considered in accordance to the distribution of the Portuguese population in each educational level in order to guarantee the real representativeness of the education of the population. National data refers to the year of 2008-9 and it is available at www.pordata.pt [20]. Weight cases were calculated as the national prevalence of education for each category divided by the education prevalence of our data. In males, the national education corresponds to a prevalence of 38.5% bellow 4 complete years of education, 38.3% 5–9 years, 14.3% 10–12 years, and 8.9% with higher education level. In females 43.9% under 4 years, 30.0% 4–9 years, 13.6% 10–12 years, and 12.5% with higher education level.

### Statistical Analysis

Data analysis was performed using IBM SPSS Statistics version 19.0, 2010 (SPSS Inc., an IBM Company, Chicago, Illinois, U.S.A.) and with MedCalc version 11.1.1.0 (MedCalc, Mariakerke, Belgium). For all tests statistical significance was set at p<0.05.

Descriptive statistics were performed to characterize the sample. Normality was tested using the Kolmogorov-Smirnov test and the Mann-Whitney Test was used for gender comparison in continuous variables. Comparison of proportions was performed with Chi-square tests. Binary logistic regression was used to analyze the association between educational level and BMI and AO. Models were developed for adults and old adults, adjusting for the age of the participants separately for gender, and for the all sample (adjusted for gender). We further verified if the association between overweight, obesity, and AO varied by sex by developing a model that included educational level, age, gender, and the interaction between gender and the educational level as independent variables.

Finally, the sample was adjusted by a weight factor, using the *weight* cases option in the SPSS software in all analysis, in order to balance the sample in accordance to educational level, and adjusted means were presented for BMI and WC, and adjusted prevalence of overweight, obesity, and abdominal obesity were also obtained considering this weight factor to guarantee the real representativeness of education levels for men and women. We further modeled our data considering the educational level reported in the Carmo et al. [9] study.

## Results

Table 1 summarizes the sample demographic data and the educational level.

**Table 1:** Descriptive characteristics.

	18–64 years			65+ years		
	Males	Females	All Sample	Males	Females	All Sample
Sample size	3279	3179	6908	784	1755	2539
Age (yrs)	38.0±12.6	40.3±12.9^a^	39.2±12.8	75.2±7.3	75.0±7.3	75.1±7.3
Weight (kg)	78.6±11.6	63.7±10.8^a^	70.6±13.4	73.1±10.7	64.7±9.8^a^	67.3±10.8
Height (cm)	173.2±7.4	159.2±6.7^a^	165.7±9.9	165.0±6.9	154.3±7.4^a^	157.6±8.8
BMI (kg/m^2^)	26.2±3.7	25.2±4.5^a^	25.7±4.18	26.8±3.4	27.1±3.4^a^	27.0±3.3
WC (cm)	90.4±10.4	82.5±11.1^a^	86.2±11.4	97.9±10.4	94.5±11.9^a^	95.5±11.6
Education Level (%)						
Until 4 yrs	10.8	14.2	12.7	81.2	88.9	86.5
5–9 yrs	24.8	19.2	21.7	10.3	6.1	7.4
10–12 yrs	30.6	27.5	29.0	4.3	2.1	2.8
College	33.8	39.0	36.6	4.2	2.9	3.3

Abbreviations: BMI, body mass index; WC, waist circumference.

a Significant differences between gender (p<0.05).

Overall, in the adult population, females presented lower BMI and WC than males (p<0.05). Old female adults presented a higher BMI, and a lower WC comparing to males (p<0.05).

The means for BMI and WC, and the prevalence of overweight, obesity, and AO, by gender and age groups are presented in Table 2. The proportion of participants in each age group and gender, and the distribution by geographical areas closely follows the official demographic data for Portugal. However, due to the education discrepancy when comparing to National official data, all means and prevalence reported in Table 2 are adjusted for the educational level weight factor.

**Table 2:** Body mass index and waist circumference and prevalence of overweight, obesity and abdominal obesity in the adult population of Portugal, by sex and age category, adjusted for educational level.

	Sample size	BMI (kg/m^2^)	Overweight (%)	Obesity (%)	WC (cm)	Abdominal Obesity (%)
**Males**						
**Adult**						
18–24 years	364	24.1±3.3	26.9	4.4	84.0±8.8	4.3
25–34 years	726	25.7±3.4	42.0	11.4	87.9±9.1	8.4
35–44 years	643	27.1±3.8	47.2	21.6	92.8±10.4	18.6
45–54 years	778	27.9±3.9	52.7	27.4	96.1±10.5	26.5
55–64 years	668	28.3±3.7	55.1	27.4	97.6±10.1	31.7
**All**	**3177**	**26.9±3.9** a	**46.7** a ^,^ b	**19.9**	**94.5±10.9** a ^,^ b	**19.3** a ^,^ b
**Older Adult**						
65–74 years	406	27.1±3.0	55.8	18.2	97.9±10.1	32.8
75+ years	378	26.4±3.3	51.3	15.2	97.8±10.6	31.5
**All**	**784**	**26.8±3.1**	**53.6**	**16.8**	**97.9±10.4** a	**32.1** a
**Females**						
**Adult**						
18–24 years	314	22.4±3.2	15.6	2.4	76.2±8.5	9.9
25–34 years	568	24.0±3.8	24.0	8.1	79.6±10.0	17.5
35–44 years	861	25.9±4.7	33.9	16.8	84.0±11.3	31.1
45–54 years	961	27.6±4.6	44.8	25.2	88.3±10.8	47.8
55–64 years	1025	28.2±4.4	49.8	29.0	90.0±±10.3	54.3
**All**	**3729**	**26.4±4.7** b	**38.1** b	**19.8**	**85.4±11.4** b	**37.9** b
**Older Adult**						
65–74 years	947	27.2±3.4	53.7	22.7	94.0±11.8	67.8
75+ years	808	27.0±3.3	51.9	20.7	95.1±12.1	71.9
**All**	**1755**	**27.1±3.4**	**52.9**	**21.8**	**94.5±11.9**	**69.7**

Abbreviations: BMI, body mass index; WC, waist circumference.

Results presented are adjusted for the weight factor for educational level.

a Significant differences between gender (p<0.05).

b Significant differences between adult and older adults (p<0.05).

In both adults and old adults the proportion of participants with normal weight was less than 50%.

Considering the participants aged 18–64 years, 66.6% of males were overweight (46.7%) or obese (19.9%). In females, the prevalence of overweight was 38.1%, while 19.8% of females were obese. Overall, these results correspond to 57.9% of female adults with overweight or obesity. We also observed that the prevalence of AO was higher in adult females (37.9%) when comparing to males (19.3%).

In both genders, old adults' prevalence of overweight (males: 53.6%; females: 52.9%) and abdominal obesity (males: 32.1%; females: 69.7%) was higher than in adults. However, no differences were observed between age groups when comparing obesity prevalence (old adults, males: 16.8%; females: 19.8%). In participants aged 65 and older, gender differences in obesity prevalence were only observed for AO, with females presenting a higher proportion compared to males.

In both genders of adults, the higher prevalence of overweight, obesity, and AO was observed at older ages. In older adults this trend was reversed. At the age group of 75+ a lower prevalence was observed comparing to the 65–74 years age category, except for AO in females.

**Figure 1:**
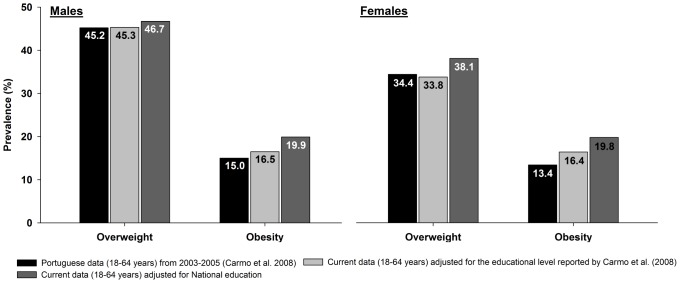
Prevalence of overweight and obesity in Portuguese adult population. Data from the current study, adjusted for national education and for the education reported in the 2003–2005 survey, and prevalence from the 2003–2005 survey in the adult population.

Figure 1, presents (for participants aged 18–64 years), a comparisons of the prevalence of overweight and obesity for our sample, adjusted for the weight factor for educational level [20] ; the adjusted for the educational level prevalence reported at the 2003–2005 survey [9], and the prevalence data from the 2003–2005 survey reported by Carmo et al. (2008), for the adult population. Considering the educational level reported at the 2003–2005 survey [9], in males, the educational level corresponds to a prevalence of 21.3% under 4 years, 21.0% 5–9 years, 31.6% 10–12 years, and 26.1% with college education. In females 24.5% under 4 years, 17.5% 4–9 years, 31.4% 10–12 years, and 26.5% with higher education level.

**Figure 2:**
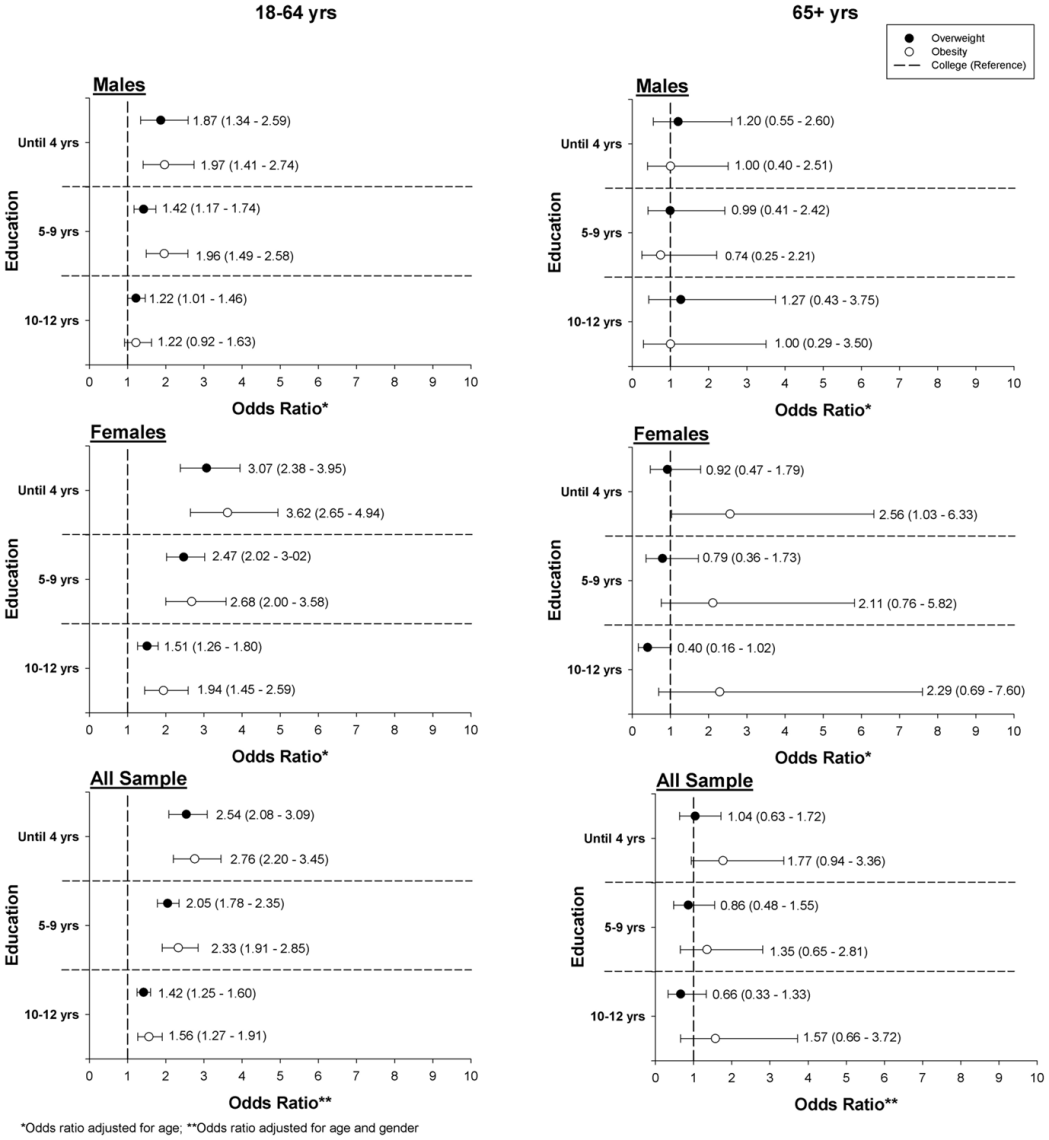
Odds-ratio for overweight and obesity according to the educational level, for adults and old adults.

For subjects aged 18–64 years there was an association between the educational level and the odds-ratio (OR) for overweight and obesity, as shown in Figure 2. For the all sample, participants with 4 years, 5–9 years, and 10–12 years of education presented increased OR for overweight, and obesity considering college education as reference. The higher OR were found for adults with the lower education, with 4 years or less (males: OR for overweight was 1.87 and OR for obesity was 1.97; females: OR for overweight was 3.07 and OR for obesity was 3.62). We further verified that the associations with the educational level varied by gender for overweight (≤4 years and 5–9 years of education) and obesity (≤4 years and 10–12 years of education) as the interaction terms between gender and educational level were significant predictors in the model. Considering older adults, a higher OR for obesity was observed for females with the lower education (OR = 2.56). No differences on the association with educational level were observed between genders for overweight and obesity, as the interaction terms between gender and educational level were not significant in the model.

**Figure 3:**
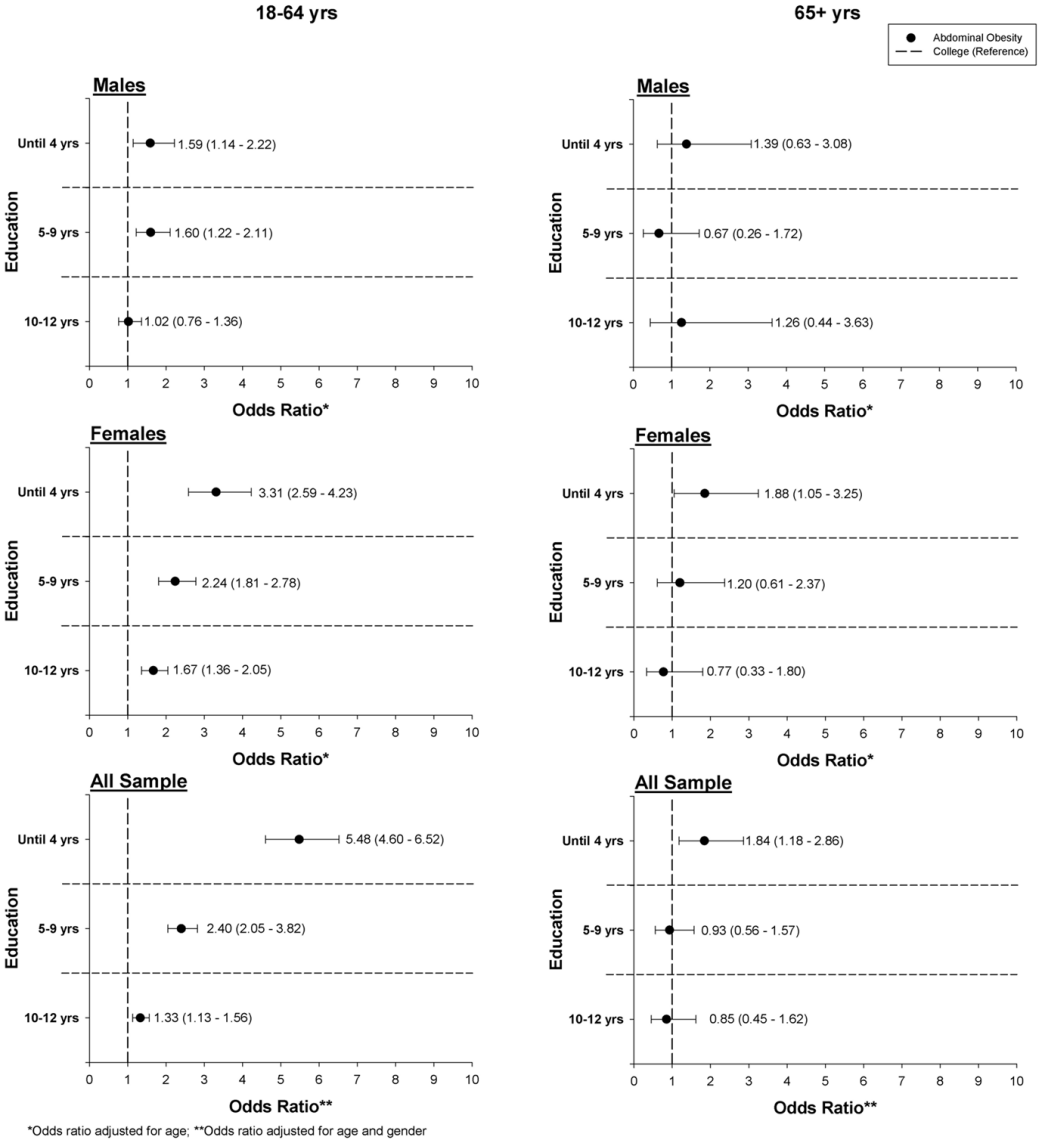
Odds-ratio for abdominal obesity according to the educational level, for adults and old adults.

In adults, a significant association between educational level and the OR for AO was also found (p<0.05), as observed in Figure 3. Participants with 4 years (males: OR = 1.59; females: OR = 3.31), 5–9 years (males: OR = 1.60; females: OR = 2.24), and 10–12 years (males: OR = 1.02; females: OR = 1.67) of education presented increased OR for AO. The association with the educational level varied by sex (≤4 years and 10–12 years of education), as the interaction terms between gender and educational level were significant predictors in the model.

In old adults (Figure 3), a significant increased OR for AO was found for females (1.88) and for both genders (1.84) with 4 years of education. No differences on the association with educational level were found between genders.

## Discussion

The current investigation aimed to characterize the current prevalence of overweight, obesity, and abdominal obesity of the Portuguese adult population (aged 18 and older). For the first time in Portugal this study provides adult prevalence data with objectively measured weight and height and WC adjusting for population educational level guarantying the real representativeness of the education levels of this population.

Overall, in the adult population (18–64 years), the prevalence of overweight in men was 46.7% while women presented a lower prevalence (38.1%) and the proportion of adults with obesity is near 20% for both genders. With data collected five years earlier, Carmo et al. [9], in the same age range (18–64 years) observed that the prevalence of overweight and obesity in Portugal was below of the one now reported, though the author's results were not adjusted for educational level. Indeed, the fact that the sample in the Carmo et al. [9] study had a higher education level than the Portuguese population [20], may partly explain these differences. For comparative purposes, we modeled our data to the educational level reported in the Carmo et al. [9] study. In the five-year period difference of data collection between both studies, we can estimate that there was a slight increase in male obesity (1.5%) and in female obesity (3.0%). A recent investigation [8] analyzed a possible leveling off of the obesity in the last years. Although stability or a leveling off in the prevalence of obesity is evident among children and adolescents, in adults the tendencies are mixed when looking at data from different countries. Nevertheless, the level of prevalence of overweight and obesity worldwide is higher than ever before and there are reasons to be concerned with the future in some populations [8]. In Portugal, a systematic review using data obtained from anthropometric measures, observed that the prevalence of overweight and obesity altogether has evidenced a modest increase in older subjects, from 1995 to 2005 [10].

In our study we also included the population aged 65 and older, and observed that they presented higher prevalence of overweight comparing to the adult sample (18–64 years). In old adults the prevalence of overweight was 53.6% and 52.9% for males and females, respectively, while 16.8% of men and 21.8% of women were obese. With self-reported weight and height collected in 2005-6, Marques-Vidal et al. [11], used the same age range (18+ years) and the same age interval than the one in this investigation. Consistent with the present study, the authors also observed that the prevalence of overweight was higher at older ages, with the exception of the 65–74 to the 75+ years age group, where a lower BMI was observed. Conversely, when we compared our prevalence of overweight and obesity with the 2005-6 data reported in this study [11], we observed, for all age groups and gender, higher BMI, and consequently higher prevalence of overweight and obesity for the Portuguese population. However, the author used self-reported measures, and it has been shown that subjects tend to underestimated their weight and to overestimated their height, resulting in underestimation of BMI when using self reported measures [12], [13].

In general, the current investigation observed that more than two thirds of the Portuguese adult population aged 18 and older, are overweight or obese. Thus, there is the need for effective interventions, with population-based approaches to prevent and treat obesity. In comparison to recent data (2000–2009) from other European countries [21] that used measured height and weight to calculate BMI, the Portuguese population, as assessed in the current study, presents a lower prevalence of overweight in the adult population (25–64 years). With the exception of Germany, all the quoted countries had higher prevalence of obesity. Recent data from Spain [22] reported lower prevalence of overweight. However, considerable higher prevalence of obesity was reported. Observing data from countries from other continents, in Portugal the prevalence of overweight and obese adult population is considerably higher than the reported for Mozambique [23]. In this country only 18.6% of females and 11.7% of males presented BMI above or equal to 25 kg/m^2^. Also China presents lower prevalence of overweight and obesity [24]. In opposite the prevalence of subjects with overweight and obesity in the USA [25] is higher than the one we reported.

The current investigation also aimed to characterize the prevalence of AO of the Portuguese population. WC assumes greater value as a marker of abdominal visceral fat and recent investigations have verified that higher WC, independent of BMI, is associated with an increased risk for cardiovascular diseases and all-cause mortality in adults and old adults [26], [27], [28]. In the present study we observed that females have a higher prevalence of abdominal obesity than men (19.3% and 37.9% for males and females, respectively). In the sample aged 65 and older, higher prevalence was observed comparing to the adult sample (18–64 years). In old adults, similarly to the adult population, females presented higher prevalence (69.7%) when comparing to males (32.1%). Carmo et al. [9] also characterized AO of the Portuguese population, however the authors used the WHO measuring protocol [29] to measure WC circumference and in the current investigation we considered the NIH guidelines [18]. It has been suggested that different protocols may lead to different WC values, particularly among females, where the difference between the NIH and the WHO protocol is about 5 cm, leading to a prevalence of elevated WC 14% higher with the NIH procedure [30] and thus it is not possible to compare our results with those reported earlier by Carmo et al. [9].

Prior research has demonstrated that lower socio-demographic level is related to poorer health behaviors [31]. Studies in Europe and in the USA have verified that educational inequalities are related with increased BMI, with people with lower educational attainment being more likely to be overweight or obese [14], [15], and this occurs more markedly in younger adults and in females. In Portugal, Marques-Vidal et al. [11] observed that the prevalence of overweight and obesity in the Portuguese population were higher in subjects with low education, when comparing to men and women with university level. In the current study we observed that adults with a lower educational level had higher risk for overweight, obesity, and AO, reflected in the increased OR of lower educational levels when comparing to college education. Nevertheless, in accordance to other investigations [14], the higher OR for overweight (3.07), for obesity (3.62), and for abdominal obesity (3.31) were observed in adult females with the lower educational level (4 years of education or less). Our results further support assertions that strategies and campaigns need to be scientifically based on the nature of these relationships in order to target those that are more uncovered to obesity [32]. Indeed, recently it has been calculated that health inequalities in the European Union have substantial economic costs. Improving the health of those in the bottom half of the health distribution would save about 20% of the overall health care budget, about 15% of social security benefits and enhance labor productivity to the extent of about 1.4% of gross domestic product [33].

In conclusion, we observed that more than two thirds of the Portuguese population is overweight or obese. In adults (18–64 years) 66.6% and 57.9% of males and females, respectively presented BMI≥25 kg/m^2^, and in old adults (≥65 years) the prevalence is higher with 70.4% of males and 74.7% of females being overweight or obese. The association of low educational level with risk for total body overweight, obesity, and AO found in this study reinforces the importance of developing strategies to address literacy inequalities and to improve the educational level of the population in order to prevent obesity and its health complications.
